# Regreening properties of the soil slow-mobile H_2_bpcd/Fe^3+^ complex: Steps forward to the development of a new environmentally friendly Fe fertilizer

**DOI:** 10.3389/fpls.2022.964088

**Published:** 2022-08-04

**Authors:** Fabio Piccinelli, Davide Sega, Andrea Melchior, Silvia Ruggieri, Martina Sanadar, Zeno Varanini, Anita Zamboni

**Affiliations:** ^1^Department of Biotechnology, University of Verona, Verona, Italy; ^2^Dipartimento Politecnico di Ingegneria e Architettura, University of Udine, Udine, Italy

**Keywords:** cucumber, Fe-chelate, Fe leaching, N,N′-bis(2-pyridylmethyl)-*trans*-1,2-diaminocyclohexane N,N′-diacetate, plant regreening

## Abstract

The application of synthetic Fe-chelates stands for the most established agronomical practice to alleviate lime-induced chlorosis, which still constitutes a major agronomic problem. However, the percolation through the soil profile due to the negative charge of the most deployed molecules results in agronomical and environmental problems. H_2_bpcd/Fe^3+^ complex features distinctive chemical characteristics, including moderate stability of the Fe(bpcd)^+^ species (log*β_*ML*_* = 20.86) and a total positive charge, and we studied its behavior in soil and regreening effects on cucumber plants. Soil column experiments have underlined that H_2_bpcd/Fe^3+^ is retained in more amounts than EDDHA/Fe^3+^. The new ligand was not proven to be toxic for the cucumber and maize seedlings. A concentration of 20 μM H_2_bpcd/Fe^3+^ attained regreening of Fe-deficient cucumber plants grown in the hydroponic solution supplied with CaCO_3,_ similar to that shown by EDDHA/Fe^3+^. Experiments with a 2 μM concentration of ^57^Fe showed that cucumber roots absorbed H_2_bpcd/^57^Fe^3+^ at a slower rate than EDTA/^57^Fe^3+^. The high kinetic inertness of H_2_bpcd/Fe^3+^ may explain such behavior.

## Introduction

**FIGURE 1:**
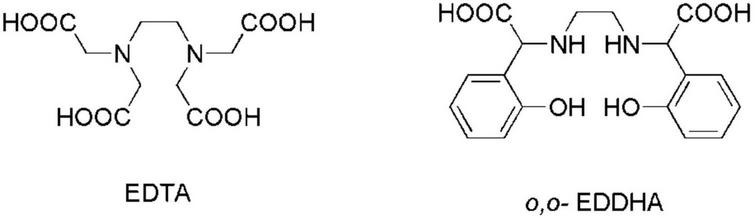
Molecular structures of EDTA and *o*,*o*-EDDHA.

Among all metal micronutrients, iron (Fe) is a metal that is required in the largest amount by plants and plays a key role in the fundamental metabolic processes, such as respiration, photosynthesis, and chlorophyll biosynthesis (Broadley et al., 2012). Albeit its relatively high abundance in the Earth’s cultivated soils, Fe bioavailability is often scarce, particularly in calcareous soils, which cover over 30% of the Earth’s land surface (Chen and Barak, 1982). The alleviation of Fe chlorosis and lime-induced chlorosis still stands as a major agronomic problem. The main approaches to alleviate Fe deficiency include the increase of soil’s indigenous Fe availability, the improvement of plant efficiency in Fe uptake and translocation, and the supply to plants with external sources of Fe (Shenker and Chen, 2005). This last technique is the most widely used. Fe fertilizers are commonly applied either to the soil or to the foliage. Fe fertilizers comprise Fe inorganic salts, synthetic Fe^3+^-chelates (synthetic products of medium-high stability), and a selected number of Fe complexes with low stability (López-Rayo et al., 2019). The usage of Fe salts is deterred by their scarce uptake in leaves, further re-translocation, and rapid precipitation under neutral–alkaline pH conditions featuring in calcareous soils. Though the usage of nanosized materials has recently changed the view about the application of Fe salts to plants (Sánchez-Alcalá et al., 2012; Sega et al., 2019), their adoption is yet to be widespread. Since the 1950s, plants have been treated with Fe^3+^-chelates (Weinstein et al., 1954; Hill-Cottingham and Lloyd-Jones, 1957). A chelate consists of a hybrid organic–inorganic molecule that is formed when a polydentate ligand bonds to a central metal ion. Fe-chelating agents used in agriculture are generally derived from polyaminocarboxylic acids. Well-known examples feature ethylenediamine tetraacetic acid (EDTA) and ethylenediamine-N,N′-bis(o-hydroxyphenylacetic) acid (*o*,*o*-EDDHA) (Figure 1), with the latter being the most effective even in calcareous soils (Lucena, 2003; Abadía et al., 2011).

However, these molecules present several problems: percolation through the soil profile due to their total negative charge, little biodegradability, high cost, and synthesis of by-products whose identity is sometimes unknown. Their noteworthy scarce residual effect often demands repeated treatments, thereby not representing a sustainable solution for Fe chlorosis in plants (Rombolà and Tagliavini, 2006). Hence, the number of studies regarding the synthesis of new ligands and application in plant Fe nutrition has increased in the last 5 years (Santos et al., 2016; Martín-Fernandez et al., 2017; López-Rayo et al., 2019; Valverde et al., 2021).

Engineering a Fe-chelate to act as a reservoir can overcome the percolation issues, since a slow-release fertilizer increases the presence of Fe in the soil solution in the long term. This feature is strongly related to the sorption mechanisms of Fe-chelates on soil surfaces, which has been well-reviewed by Lucena (2006). A slow Fe release seems possible whenever the chelate features moderate stability allowing it to “react” with the treated soil. Indeed, less stable chelates can lose some of their links with Fe^3+^ more easily (i.e., the ones with the carboxyl groups) and subsequently interact with the soil surface (Hernández-Apaolaza and Lucena, 2001). While the highly stable *o,o*-EDDHA/Fe^3+^ chelate (log*β_*ML*_* = 35.09)^1^ (Yunta et al., 2003) interacts weakly with the soil and concurrently lies in the soil solution in high concentrations, the less stable EDTA/Fe^3+^ chelate (log*β_*ML*_* = 25.1) represents a more ideal candidate. Nevertheless, this chelate is not able to hold the Fe^3+^ ion available to the plant in calcareous soils, in which all the Fe precipitates as Fe(OH)_3_ due to the Ca displacement from the Fe-chelate (Lucena, 2006).

**FIGURE 2:**
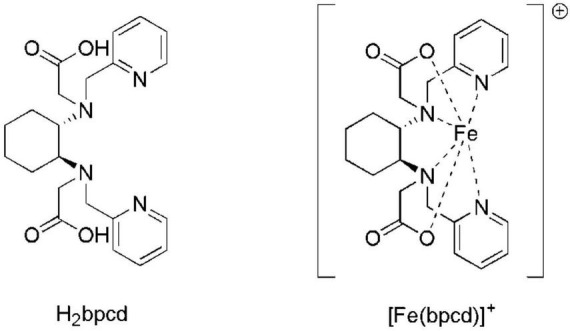
Molecular structures of H_2_bpcd and [Fe(bpcd)]^+^, the molecules synthesized and discussed in the present contribution. The racemic form of the ligand has been employed.

In this work, we propose a new Fe-chelate based on the racemic form of the ligand N,N′-bis(2-pyridylmethyl)-*trans*-1,2-diaminocyclohexane N,N′-diacetate (H_2_bpcd; Figure 2) as Fe-EDTA-like molecule capable to interact effectively with the soil, thanks to both its moderate stability (log*β_*ML*_* = 20.86) and a total positive charge. Hence, it is expected that [Fe(bpcd)]^+^ should be strongly retained by the negatively charged soil particles. These particles prevail in neutral alkaline soils, thereby enabling a progressive Fe release at the rhizosphere *via* desorption and/or Fe^3+^ reduction (Strategy I plants) or ligand exchange (Strategy II plants). Therefore, we studied the behavior and performance of [Fe(bpcd)]^+^ in simplified systems, such as soil columns and hydroponic culture.

## Materials and methods

### N,N′-bis(2-pyridylmethyl)-*trans*-1,2-diaminocyclohexane N,N′-diacetate (H_2_bpcd) synthesis

(±)-N,N′-bis(2-pyridylmethyl)-*trans*-1,2-diaminocyclohexane N,N′-diacetate (H_2_bpcd) was synthesized as described by a previous study (Oddon et al., 2012).

### Synthesis of the Fe^3+^ complex, H_2_bpcd/Fe^3+^

H_2_bpcd trifluoroacetate salt (300 mg, 0.7278 mmol) was dissolved in ethanol (60 mL) under an inert atmosphere (Ar). FeCl_3_⋅6H_2_O (196.7 mg, 1 eq) was added to the ligand solution under vigorous stirring. Then, potassium acetate (357.2 mg, 5 eq) dissolved in 30 mL of ethanol was added to the solution. The solution was left for 30 min at 95°C under reflux and later was allowed to cool down. The solution was stored for 2 h at −20°C and then centrifuged at 4,500 rcf for 15 min at 0°C, to separate the inorganic salts. The resulting supernatant was dried under a vacuum. The obtained brownish solid was triturated with diethyl ether several times to remove all of the residual trifluoroacetic acid and water. The resulting yellowish solid was left overnight under a vacuum. The obtained product weighed 467.7 mg with a yield of 82%. Elemental Anal. Calc. for C_24_H_29_FeN_4_O_6_ (MW 525,36): C, 54,87; H, 5.56; N, 10.66; O, 18.27. Found C, 54.20; H, 5.48; N, 10.50; O, 18.52. ESI-MS (Scan ES+; *m/z*) in methanol: 520.30 (100%), 521.25 (26%); [Fe(bpcd) (OCH_3_)Na]^+^; 498.47 (100%), 499.14 (26%); [Fe(bpcd) (OCH_3_)H]^+^.

In the case of the synthesis of H_2_bpcd/^57^Fe^3+^, a slight excess of 6M hydrochloric acid (HCl) was added to ^57^Fe(III) oxide(^57^Fe_2_O_3_) to produce ^57^Fe(III) chloride (^57^FeCl_3_) according to the following reaction:


FeO2+36HCl→2FeCl+33HO2

The solution was then heated slightly to eliminate excess HCl. Then the compound was freeze-dried for a day to remove the excess water, and ^57^FeCl_3_⋅2H_2_O was finally isolated. To obtain [^57^Fe(bpcd)](CH_3_COO) complex, we used the same synthetic protocol adopted for [Fe(bpcd)](CH_3_COO) molecule. A similar chemical yield was obtained. The complex was analyzed by ICP-MS to determine the purity (82.4% of [^57^Fe(bpcd)](CH_3_COO) and 17.6% of potassium acetate).

### Synthesis of EDTA/^57^Fe^3+^

EDTA/^57^Fe^3+^ was synthesized as described by Loots et al. (2007).

### Speciation in solution

The standard solutions of Fe^3+^were prepared by dissolving the required amount of chloride salt (Sigma) in Milli-Q water and adding a stoichiometric amount of HCl to prevent hydrolysis. The NaOH and HCl stock solutions were prepared with Fixanal (Fluka Analytical) and Milli-Q water (>18 MΩ cm) in a Milli-Q system (ELGA Purelab UHQ). The Fe^3+^ stock solution was standardized by spectrophotometric determination of the DFO (Desferal) complex (Cappai et al., 2018). Spectrophotometric data were collected (220–360 nm) for a series of solutions in the pH range of 0–2. Spectrophotometric data were analyzed using the HypSpec program (Gans et al., 1996). Ligand protonation constants were taken from Leonzio et al. (2017) and kept constant during the refinement procedure.

The potentiometric data were also collected at pH > 2 to determine the presence of additional equilibrium. Before each titration, the electrode was calibrated by acid–base titrations with standard HCl and NaOH solutions. Electromotive force (emf) values were measured using a combined glass electrode (MetrohmUnitrode 6.0259.100) and collected by a computer-controlled device (Amel Instruments, 338 pH Meter). The temperature in the titration cell was maintained at 25.0 ± 0.1 °C by means of a circulatory bath. Carbon dioxide content in the solution was checked by Gran’s method (Gran, 1952). Complex formation constants were obtained by processing the experimental data with the Hyperquad program (Gans et al., 1996). Hydrolysis constants for Fe^3+^ at *T* = 25°C and *I* = 0.1 M (NaCl) were taken from the literature (Stefánsson, 2007). Speciation calculations were done by means of the software ChemEQL v3.2^2^. The speciation model regarded the ligand protonation and metal (Fe^3+^, Ca^2+^) complex formation constant; they were determined in this work or taken from the literature (Lacoste et al., 1965; Delgado et al., 1997; Lucena, 2003). The model comprised the presence of a CaCO_3_ solid phase (calcite). Equilibrium constants for metal hydrolysis, hydroxide precipitation, carbonate acid–base, and complexation equilibria for the speciation model were also taken from the literature (Stefánsson, 2007) and the internal databases of ChemEQL (Smith and Martell, 1976; Morel and Hering, 1993; Stumm and Morgan, 1995).

### HPLC analysis

The [(Fe(bpcd)](CH_3_COO) complex was analyzed with a Shimadzu HPLC, using a C18, 00G-4053-E0 Jupiter is: 5 μM 300 Å New column 250 × 4.6 mm^2^ 671,529-9. The mobile phases were H_2_0 + 0.1% TFA (phase A) and ACN + 0.1% TFA (phase B). Mobile phase flux constituted 1 mL min^–1^ with a ramping from 5% phase B to 95% phase B within 20 min, and 95% phase B was kept until 25 min. The acquired wavelengths were 300, 280, 260, 263, 265, and 250 nm. The retention time of the complex was 23 min (Supplementary Figure 1), and no extra peaks belonging to impurity have been detected.

### Elemental analysis

Elemental analyses were carried out by using an EACE 1110 CHNOS analyzer.

### ESI-MS

Complex purity was assessed *via* ESI-MS analysis of H_2_bpcd [(Fe(bpcd)^+^(CH_3_COO) ^–^] performed with a Finnigan LXQ Linear Ion Trap (ThermoScientific, San Jose, CA, United States) and an electrospray ionization source (ESI) operating in positive ion mode. Data acquisition was performed with Xcalibur software (Thermo Scientific). H_2_bpcd and [(Fe(bpcd)^+^(CH_3_COO) ^–^] were dissolved in methanol and injected into the ion source at a rate of 10 μL min^–1^, with the temperature of the transfer line being 275 °C and 4.70 kV He pressure in the ionic trap was 1 mTorr.

### Soil leaching experiments

The study of the interaction between H_2_bpcd/Fe^3+^ and soil particles was carried out using a soil column. A silt-loam soil was utilized, with the following chemical characteristics: pH (in water) 7.0, organic matter 2.21%, C.E.C. 23.7 meq/100 g, total limestone 10.9%, active limestone 2.3%, organic carbon 1.28%, total nitrogen 0.15%, available phosphorous 3.9 mg/kg, soluble boron 0.09 mg/kg, and C/N ratio 8.8.

A glass column (height: 43 cm, diameter: 4 cm, and soil weight: 284.61 g) was filled with soil as follows: the dry soil was sieved at 2 mm, loaded into the column from above and pounded to obtain a homogeneous profile, and the column was then supersaturated from above with deionized water. A 1-cm layer of silica sand was put on top of the column to avoid column digging. One milliliter of 7.7 mM Fe complex (EDDHA/Fe^3+^ or H_2_bpcd/Fe^3+^) solution (7.7 μmol of total Fe) was applied to the column and eluted with Milli-Q water. After a dead volume leakage of 50 mL, the eluate was collected in 5-mL fractions. Elution fractions were used to quantify Fe by ICP-MS analysis. The soil leaching experiment was also conducted with a 0.5 M CaCl_2_ solution for the elution instead of water.

### Phytotoxicity test

To evaluate the potential toxicity of H_2_bpcd for plants, germination inhibition and root seedling elongation were evaluated for *Cucumis sativus* (Viridis F1 hybrid seeds) and *Zea mays* (inbred line P0423, Pioneer). Forty seeds of *Cucumis sativus* and 45 seeds of *Zea mays* were germinated in boxes (23.50 × 18 cm^2^ for cucumber; 24.50 × 30 cm^2^ for maize) on filter paper moistened with a solution (20 mL for cucumber; 32 mL for maize) of H_2_bpcd or NaEDTA at the following concentrations: 0, 10, 50, and 100 μM. The boxes were closed with plastic film and aluminum foil and incubated at 25°C in the dark. Germination time consisted of 4 days for cucumber and 3 days for maize. The experiment was repeated four times.

### Plant materials and treatments

Cucumber seeds (*Cucumis sativus* L. and Viridis F1 hybrid) were germinated for 6 days in a closed box with the filter paper moistened with 1 mM CaSO_4_ solution. The seeds were incubated in a dark chamber at 25°C. Growth experiments were carried out in hydroponics. After the germination, six seedlings were grown for 7 days in one black pot with 1.8 L of nutrient solution featuring the following composition: 0.7 mM K_2_SO_4_, 2 mM Ca(NO_3_)_2_, 0.5 mM MgSO_4_, 0.1 mM KH_2_PO_4_, 0.1 mM KCl, 10 μM H_3_BO_3_, 0.5 μM MnSO_4_, 0.5 μM ZnSO_4_, 0.2 μM CuSO_4_, and 0.01 μM (NH_4_)_6_Mo_7_O_24_ in the presence of 1 g/L CaCO_3_. Fe as Fe(EDDHA) with the final concentration equal to 50 μM (positive control) was added purposely to one pot. The growth parameters were as follows: temperature of 25°C, 16/8 h light/dark regime, relative humidity of 50%, and light intensity of 200–250 μmol m^–2^ s^–1^ PPFD (photosynthetic photon flux density). The nutrient solution was kept aerated by means of a pump system. After 7 days, the Fe-deficient seedings (previously grown without Fe) were supplied with the aforementioned nutrient solution at three different concentrations (0.2, 2, and 20 μM) of two Fe sources, such as Fe(bpcd)]^+^ and Fe(EDDHA). One pot (six seedlings) was maintained without Fe (negative control). The positive control was grown for further 7 days in similar conditions. Three growth and treatment experiments (biological replicates) with six plants each (technical replicates) were independently repeated, for a total of 18 plants.

The fresh and dry weight of shoots and roots, and the leaf count were determined for each plant. In addition, soil and plant analyzer development (SPAD) chlorophyll indices were measured by using a chlorophyll meter (Konica-Minolta). The average size of each leaf was determined based on five measurements. Throughout each experiment, shoots and roots of three plants per each condition were washed for three times with Milli-Q water. Thereafter, tissue samples were dried for 3 days at 60°C, homogeneously ground with mortar and pestle, and used for the determination of macro- and micronutrient content by ICP-MS analysis.

The root apparatus was scanned with an Epson WIA scanner. Acquired images were then analyzed with WinRHIZO™ software, 2015a Pro version (Regent Instruments Inc.), using the “root morphology” mode.

### Activity of ferric-chelate reductase

Three plant roots were washed for three times with Milli-Q water to remove any residues of nutrient solution. The root apparatus was incubated in a 0.1 mM disodium EDTA (Na_2_EDTA) solution to remove chelate-free and apoplastic Fe. The roots were then washed three times with Milli-Q water to remove EDTA and then incubated in 20 mL of assay solution for 20 min at 25°C in the darkness. The assay solution had the following composition: 0.5 mM CaSO_4_, 500 μM 3-(2-pyridyl)-5,6-bis(4-phenylsulfonic acid)-1,2,4-triazine also called ferrozine (PDT), 250 μM ferric sodium EDTA (FeNaEDTA), 10 mM 2-(*N*-morpholino) ethanesulfonic acid sodium hydroxide (MES NaOH), and pH 6.0. The formation of the complex between chromophore PDT and its ligand Fe^2+^, produced by the root ferric-chelate reductase (FCR) activity, was determined spectrophotometrically by measuring the absorbance at 562 nm (Stookey, 1970). The activity of the enzyme was calculated as nmol Fe^2+^g^–1^ root FW h^–1^.

### ^57^Fe absorption experiments

The cucumber seeds were germinated as previously described and hydroponically grown in pots with nutrient solution for only 7 days, without the addition of any Fe source. The composition of the nutrient solution was the same as detailed in section “Plant Materials and Treatments” but without the addition of CaCO_3_. Two assay solutions for the ^57^Fe absorption experiment were prepared by using the nutrient solution with H_2_bpcd/^57^Fe^3+^ or EDTA/^57^Fe^3+^, with the concentration of Fe reaching 2 μM. The root apparatus of each plant was incubated in 50 mL of the assay solution. The experiment was performed at 25°C by maintaining the root apparatus in the dark. The nutrient solution was kept aerated by means of a pump system. The assay was carried out for 1 and 24 h. The assay was performed for both H_2_bpcd/^57^Fe^3+^ and EDTA/^57^Fe^3+^ chelates. The root apparatus of each cucumber plant was washed with Milli-Q water and incubated in the assay solution. After 1 or 24 h, the roots were sequentially washed once with a 0.1 mM Na_2_EDTA solution and twice with Milli-Q water. Samples were dried for 3 days at 60°C and homogeneously ground with mortar and pestle. Thereafter, they were digested and analyzed at ICP-MS.

### ICP-MS analysis

The ICP-MS analysis was used to establish the Fe concentration of each fraction eluted from the columns in the soil leaching experiment and the macro- and micronutrient content of plant tissues. Moreover, the content of ^57^Fe in the shoots and roots was defined throughout the isotope ratio measurements carried out with the same instrument (Agilent 7500ce ICP-MS detection system, Agilent technologies). In the case of eluted fractions, samples were diluted (1:50) with 2% HNO_3_ (prepared using ultrapure 68% HNO_3_ in water and 18.2 MΩ⋅cm at 25 °C) and filtered (0.45 μm). For the analysis of the plant tissues, about 10 mg of samples was mineralized in a 3-mL TFM microsampling insert (Milestone) using 350 μL of ultrapure 68% HNO_3_. Three inserts were put into a 100-mL TFM vessel with 11 mL of Milli-Q water (18.2 MΩ⋅cm at 25°C) and 1 mL of ultrapure grade 30% H_2_O_2_. The digestion lasted for 20 min at 180°C in a StartD microwave digestion system (Milestone Srl). The digested samples were diluted up to a concentration of 2% HNO_3_ and then analyzed using an Agilent 7500ce ICP-MS detection system (Agilent technologies). Calibration curves were attained by dilution of a custom-made multielement standard (Romil LTD), with a stock solution containing K (20,000 ppm), Ca (10,000 ppm), Mg and P (2,000 ppm), Na (400 ppm), Fe (50 ppm), Mn (40 ppm), B and Zn (20 ppm), Cu (5 ppm), Co, Mo, and Se (1 ppm). Measurement accuracy and matrix effect errors were assessed using a standard reference material (NIST^®^ SRM^®^ 1515 Apple leaves). The measurement accuracy for the analysis of ^57^Fe was conducted using a certified isotopic reference material (IRMM-634).

## Results

### Speciation of H_2_bpcd/Fe^3+^ complex in aqueous solution

**FIGURE 3:**
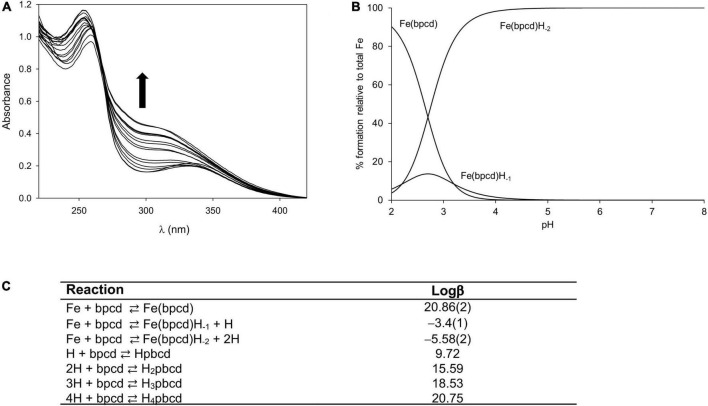
Spectral changes observed when increasing the pH from 0.0 to 2.0 in solutions containing equimolar amounts of the bpcd ligand (0.088 mM) and FeCl_3_ **(A)**. Speciation for the Fe^3+^/bpcd system (C_Fe_ = 1.0 mM, 1:1 metal/ligand molar ratio) calculated on the basis of the fitted constants in panel **(C) (B)**. Protonation (Leonzio et al., 2017) and formation constants for Fe(bpcd) species (standard deviation in parenthesis) **(C)**. Charges are omitted for clarity.

Figure 3A exhibits the spectral changes recorded during/alongside the titration of the ligand/FeCl_3_ solution from pH∼0 to 2. By increasing the pH, the maximum located at λmax = 259 nm (blue) shifts slightly (final λmax = 253 nm) and increases in intensity (Figure 3A). The fitting of spectrophotometric data at low pH provided a quite high formation constant for the 1:1 cationic complex with bpcd ([Fe(bpcd)]^+^; logβ = 20.86; Figure 3C). Protonated species [e.g., Fe(bpcd)H^+^] were discarded by the fitting program.

**FIGURE 4:**
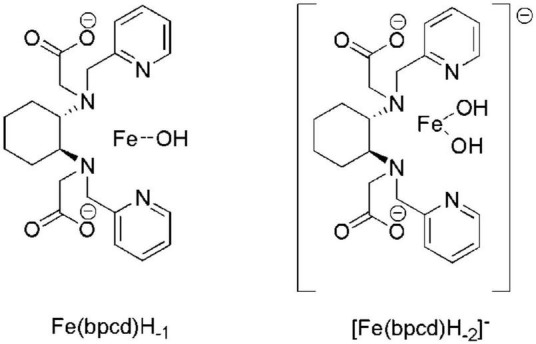
Molecular structure of the different Fe^3+^-based species observed in aqueous solution above pH 3. In Fe(bpcd)H_–1_ and [Fe(bpcd)H_–2_] ^–^ species, the ligand/Fe^3+^ dative bonds are not depicted.

Incidentally, according to the speciation derived from the formation constants herein obtained (Figure 3B), the dominant species above pH = 4 is a negatively charged hydroxo complex ([Fe(bpcd)H_–2_] ^–^; Figure 4). The speciation of *o*,*o*-EDDHA/Fe^3+^ and EDTA/Fe^3+^ as a function of the pH are shown in Supplementary Figure 2. At a level near pH 8, the dominant species are the negatively charged [Fe(EDDHA)]- (100%) and the hydroxo complex [Fe(EDTA)H_–1_]^2–^ (60%), respectively.

### Speciation of H_2_bpcd/Fe^3+^, o,o-EDDHA/Fe^3+^, and EDTA/Fe^3+^ complexes (2 μM) in solution, considering the presence of dissolved CO_2_, solid CaCO_3_, and Fe(OH)_3_

By virtue of the ChemEQL software, the speciation of the three chelates was predicted in more realistic conditions, miming those existing in calcareous soils, in which solids, such as CaCO_3_ and Fe(OH)_3_, and dissolved CO_2_ are present together with the species in the solution. The analysis of the data in Table 1 and the speciation plots of solutions containing a 2 μM total concentration of Fe^3+^ and H_2_bpcd, and *o*,*o*-EDDHA and EDTA (ligand/metal ratio = 1) in the 2-11 pH range (Supplementary Figure 3) lead to the following findings: (i) in the 2-11 pH range, we observed precipitation of Fe(OH)_3_ only in the case of EDTA ligand (above pH 5.4); (ii) at the pH recorded in our experiments in hydroponic culture (around 8), the most abundant species containing the ligand are the negatively charged bis-hydroxo complex ([Fe(bpcd)H_–2_]^–^ (10^–6^ M) (Figure 4) in the case of H_2_bpcd/Fe^3+^, the negatively charged [Fe(EDDHA)] ^–^ (2⋅10^–6^ M) in the case of *o*,*o*-EDDHA/Fe^3+^, and the negatively charged [Ca(EDTA)]^2–^ (8⋅10^–7^ M) in the case of EDTA/Fe^3+^; (iii) at pH around 8, EDTA is less effective in chelating Fe^3+^ ion, as the concentration of Fe-chelate species is by one order of magnitude lower than the other two ligands; (iv) contrary to EDTA, the less affinity for Ca^2+^ and the presence of soluble Fe-hydroxo complexes (Fe(bpcd)H_–1_ and [Fe(bpcd)H_–2_]^–^), in the case of H_2_bpcd ligand, prevented the precipitation of Fe(OH)_3_, at pH > 7.

**TABLE 1:** The most relevant species in solution at pH = 8 in the case of the investigated ligands.

Ligand	Species in solution at pH = 8 [log (molar concentration)]			
H_2_bpcd	Ca(bpcd) [−10.14]	Fe(bpcd)^+^ [−16.12] Fe(bpcd)OH [−11.52] Fe(bpcd) (OH)_2_^–^ [−5.70]	Fe(OH)^2+^ [−15.83] Fe(OH)_2_^+^ [−11.31] Fe(OH)_3_ [−10.20] Fe(OH)_4_^–^ [−11.24] Fe_2_(OH)_2_^4+^ [−30.24] Fe_3_(OH)_4_^5+^ [−39.23]	Fe^3+^ [−21.64]
*o,o*-EDDHA	Ca(EDDHA)^2–^ [−13.96] Ca(EDDHA)H^–^ [−12.48] Ca(EDDHA)H_2_ [−11.30]	Fe(EDDHA)H [−11.90] Fe(EDDHA) ^–^ [−5.70] Fe(EDDHA)OH^2–^ [−9.13]	Fe(OH)^2+^ [−16.53] Fe(OH)_2_^+^ [−12.01] Fe(OH)_3_ [−10.90] Fe(OH)_4_^–^ [−11.94] Fe_2_(OH)_2_^4+^ [−31.63] Fe_3_(OH)_4_^5+^ [−41.32]	Fe^3+^ [−22.34]
EDTA	Ca(EDTA)^2–^ [−6.09] Ca(EDTA)H^–^ [−10.91]	Fe(EDTA)H [−15.41] Fe(EDTA) ^–^ [−9.29] Fe(EDTA)OH^2–^ [−8.82] Fe(EDTA) (OH)_2_^3–^ [−8.69]	Fe(OH)^2+^ [−14.99] Fe(OH)_2_^+^ [−10.47] Fe(OH)_3_ [−9.36] (prec.) Fe(OH)_4_^–^ [−10.40] Fe_2_(OH)_2_^4+^ [−28.55] Fe_3_(OH)_4_^5+^ [−36.70]	Fe^3+^ [−20.80]

Log of molar concentration in square brackets.

### Leaching experiment in soil column

**FIGURE 5:**
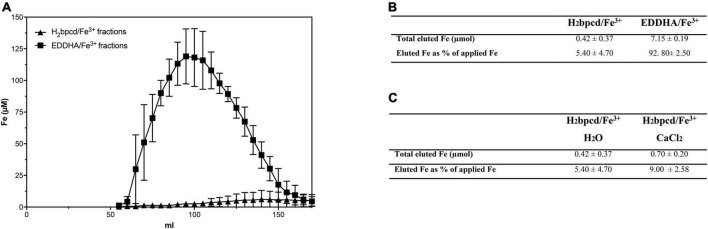
Leaching through the soil columns: **(A)** profile of eluted Fe after the application of H_2_bpcd/Fe^3+^ and EDDHA/Fe^3+^ and **(B)** comparison of the total amount of eluted Fe after the application of both Fe sources when elution was carried out with H_2_O, and **(C)** comparison of the total amount of eluted Fe after the application H_2_bpcd/Fe^3+^ between elution with H_2_O and 0.5 M CaCl_2_. Data are expressed as mean ± S.D. of three independent replicates.

The simulation of the leaching of chelates through the soil profile was carried out using a glass column filled with silt-loam soil. The amount of eluted Fe was determined by ICP-MS for each 5-mL collected fraction. As shown in Figure 5A, EDDHA/Fe^3+^ starts to exit from the column at the 65th milliliter, and at an eluted volume equal to 95 mL, Fe reaches its maximum concentration. After around 170 mL, almost all Fe loaded as EDDHA/Fe^3+^ flowed through the column. In the case of H_2_bpcd/Fe^3+^, Fe concentration in the eluate raised significantly slowly, reaching the maximum concentration at about 140 mL. By observing the amount of eluted Fe (Figure 5B), it can be inferred that almost 93% of applied EDDHA/Fe^3+^ was recovered. Conversely, the recovery of H_2_bpcd/Fe^3+^ reached only 5.4%, thereby suggesting a stronger interaction of this chelate with soil particles. H_2_bpcd/Fe^3+^ was hardly displaced from the soil even when 0.5 M CaCl_2_ occurred in the solution used for elution (Figure 5C). Only 9% of Fe was recovered at the end of the experiment.

### Phytotoxicity assays

Germination tests were performed on cucumber and maize seedlings to assess the presence of toxicity effects of H_2_bpcd ligand on plants. The results (Table 2) show that the application of H_2_bpcd or Na_2_EDTA up to 100 μM did not affect maize and cucumber germination, and morphological traits. Furthermore, they did not differ from the results of the control.

**TABLE 2:** Comparison of germination parameters for cucumber and maize seedlings when treated with different doses (10, 50, and 100 μM) of H_2_bpcd or Na_2_EDTA.

Cucumber								
	0	10 μ M		50 μ M		100 μ M		
	
	H_2_0	H_2_bpcd	Na_2_EDTA	H_2_bpcd	Na_2_EDTA	H_2_bpcd	Na_2_EDTA	p-value
% of germination	95.07 ± 5.48	97.44 ± 2.19	96.35 ± 3.11	94.34 ± 1.29	99.45 ± 1.83	97.44 ± 3.11	98.17 ± 2.37	0.275
Primary root length (cm)	7.78 ± 1.96	8.22 ± 1.88	8.02 ± 2.00	7.31 ± 2.19	8.20 ± 2.02	6.90 ± 1.88	7.77 ± 1.66	0.954
Shoot length (cm)	5.55 ± 2.38	5.61 ± 2.44	4.79 ± 2.11	4.51 ± 2.57	4.19 ± 2.05	3.89 ± 2.41	4.45 ± 2.11	0.917
**Maize**								

	**0**	**10 μ M**		**50 μ M**		**100 μ M**		
	
	**H_2_0**	**H_2_bpcd**	**Na_2_EDTA**	**H_2_bpcd**	**Na_2_EDTA**	**H_2_bpcd**	**Na_2_EDTA**	**p-value**
% of germination	93.86 ± 4.98	92.83 ± 3.28	93.80 ± 5.65	88.94 ± 6.50	95.56 ± 3.59	92.89 ± 4.13	92.83 ± 5.78	0.565
primary root length (cm)	5.91 ± 1.99	5.97 ± 2.00	5.52 ± 2.54	5.73 ± 1.70	5.53 ± 2.34	5.76 ± 1.67	5.19 ± 2.42	0.999
shoot length (cm)	1.91 ± 1.26	1.99 ± 1.37	1.59 ± 0.91	1.47 ± 1.23	1.59 ± 0.83	1.48 ± 1.18	1.51 ± 0.79	0.987

Data represent mean ± S.D. of four independent replicates (one-way ANOVA with Turkey’s post hoc test).

### Recovery of cucumber plants from Fe deficiency

**FIGURE 6:**
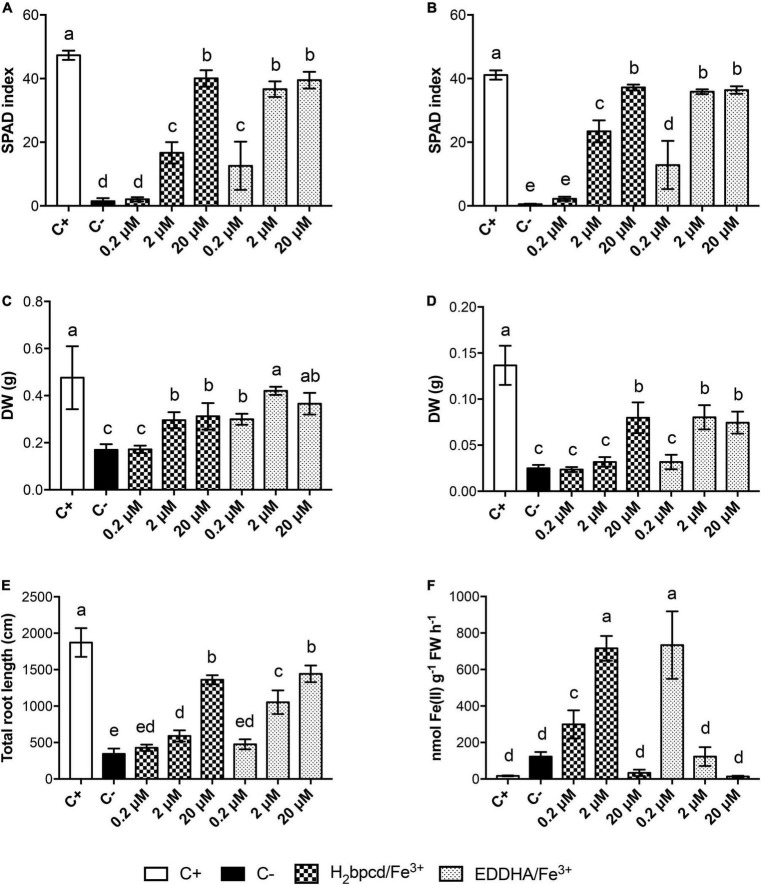
Morpho-physiological parameters of Fe-deficient cucumber plants supplied with H_2_bpcd/Fe^3+^ and EDDHA/Fe^3+^ for 7 days. **(A)** SPAD index of the first leaf, **(B)** SPAD index of the second leaf, **(C)** shoot DW, **(D)** root DW, **(E)** total root length **(F)**, and FCR activity. Data represent mean ± S.D. of three independent replicates with four plants each (*n* = 12) for panels **(A,B)** and two plants each (*n* = 6) for panels **(C–F)** (one-way ANOVA with Turkey’s *post hoc* test, *p* < 0.05, significant differences are indicated by different letters).

To assess the capability of H_2_bpcd/Fe^3+^ to supply Fe to Fe-deficient plants (Supplementary Table 1), we compared the recovery of hydroponically grown plants when supplied for 7 days with H_2_bpcd/Fe^3+^or EDDHA/Fe^3+^ at three different suboptimal concentrations (0.2 μM, 2 μM, and 20 μM) in the presence of CaCO_3_ at 1g/L. The capability of H_2_bpcd/Fe^3+^ to supply Fe was evaluated (Supplementary Figure 5) through the analysis of leaf SPAD index, root and shoot dry weight (DW), root development, and root ferric chelate reductase (FCR) activity (Figure 6).

The trend of the SPAD index was found to be quite similar in the first and the second leaves (Figures 6A,B and Supplementary Table 2). H_2_bpcd/Fe^3+^ supplied at 0.2 μM did not prompt a significant increase in the SPAD index. A partial regreening started at 2 μM of H_2_bpcd/Fe^3+^ (Figures 6A,B) and reached values similar to those of the positive control at 20 μM (40 *vs.* 47, Supplementary Table 2). EDDHA/Fe^3+^ proved to be more effective, since the regreening started even at 0.2 μM. Conversely, at the highest concentration assayed, the SPAD values did not differ significantly from those of the plants supplied with the two Fe-chelates (Supplementary Table 2). Data concerning the shoot, root biomass, and root length (Figures 6C–E) confirmed that EDDHA/Fe^3+^ can start the restoration of the plant nutritional status of Fe at concentrations lower than those of H_2_bpcd/Fe^3+^. However, even these parameters did not show significant differences at 20 μM (Supplementary Table 2).

We also measured the capacity of cucumber roots to reduce Fe. As expected, the lowest values of FCR were recorded for the positive controls (Figure 6F). The highest activity for H_2_bpcd/Fe^3+^-treated plants was recorded at 2 μM, while that of EDDHA/Fe^3+^ was found at 0.2 μM.

### Ionomic analysis

**FIGURE 7:**
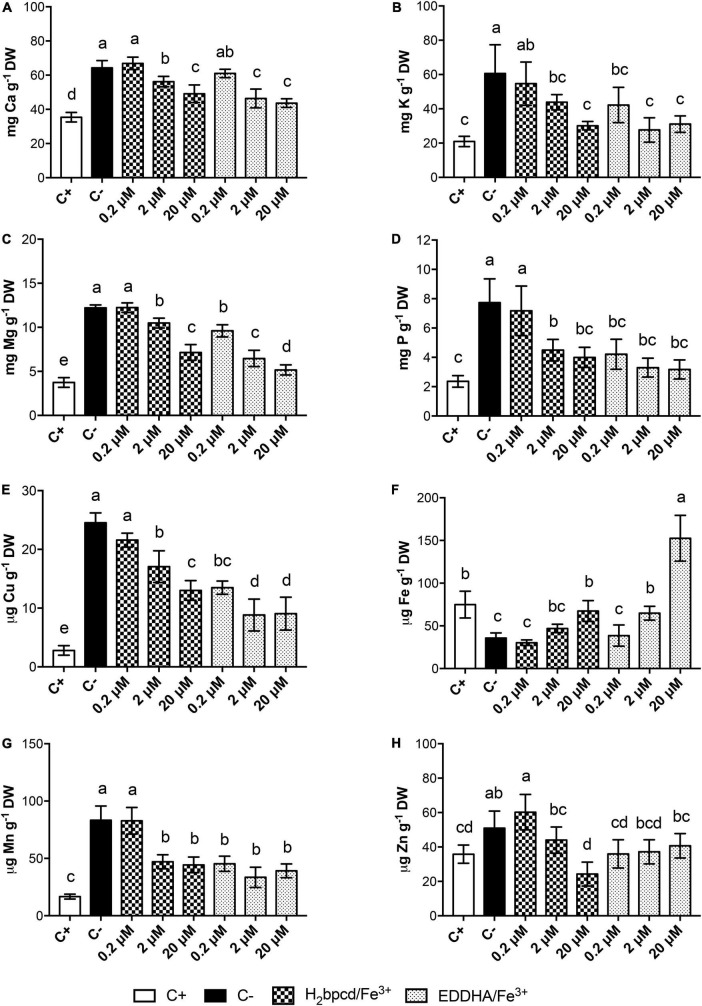
Macro- and micronutrient content in shoot tissues of Fe-deficient cucumber plants supplied with H_2_bpcd/Fe^3+^ and EDDHA/Fe^3+^ for 7 days. Shoot content of **(A)** Ca, **(B)** K, **(C)** Mg, **(D)** P, **(E)** Cu, **(F)** Fe, **(G)** Mn, and **(H)** Zn. Data represent mean ± S.D. of three independent replicates with two plants each (*n* = 6) (one-way ANOVA with Turkey’s *post hoc* test, *p* < 0.05, significant differences are indicated by different letters).

The content of Fe and other macro- and micronutrients in shoots (Figure 7 and Supplementary Table 4) and root tissues (Supplementary Tables 3, 5) was investigated by ICP-MS analysis. As foreseen, Fe-deficient plants had significantly lower Fe concentration in shoots with respect to the positive control (Figure 7F). The treatments with the two chelates exhibited trends similar to those described for the growth parameters (Figure 6). It is remarkable that the concentration of 20 μM H_2_bpcd/Fe^3+^ could restore Fe shoot content similar to the positive control, but when EDDHA/Fe^3+^ was resupplied at the same rate, the plants accumulated Fe two times as much as the positive control (Supplementary Table 4).

Data concerning other nutrients underlined that Fe deficiency determined higher content of divalent cations in shoot tissues when compared to the positive control (Figure 7).

### Short-term ^57^Fe accumulation

The capability of Fe-deficient cucumber plants to utilize the complex of H_2_bpcd with Fe was evaluated after 1 and 24 h of treatment with 2 μM H_2_bpcd/^57^Fe^3+^. The EDTA/^57^Fe^3+^ at the same concentration was used as a benchmark. The experiment was run at pH 6.0 (without CaCO_3_).

**FIGURE 8:**
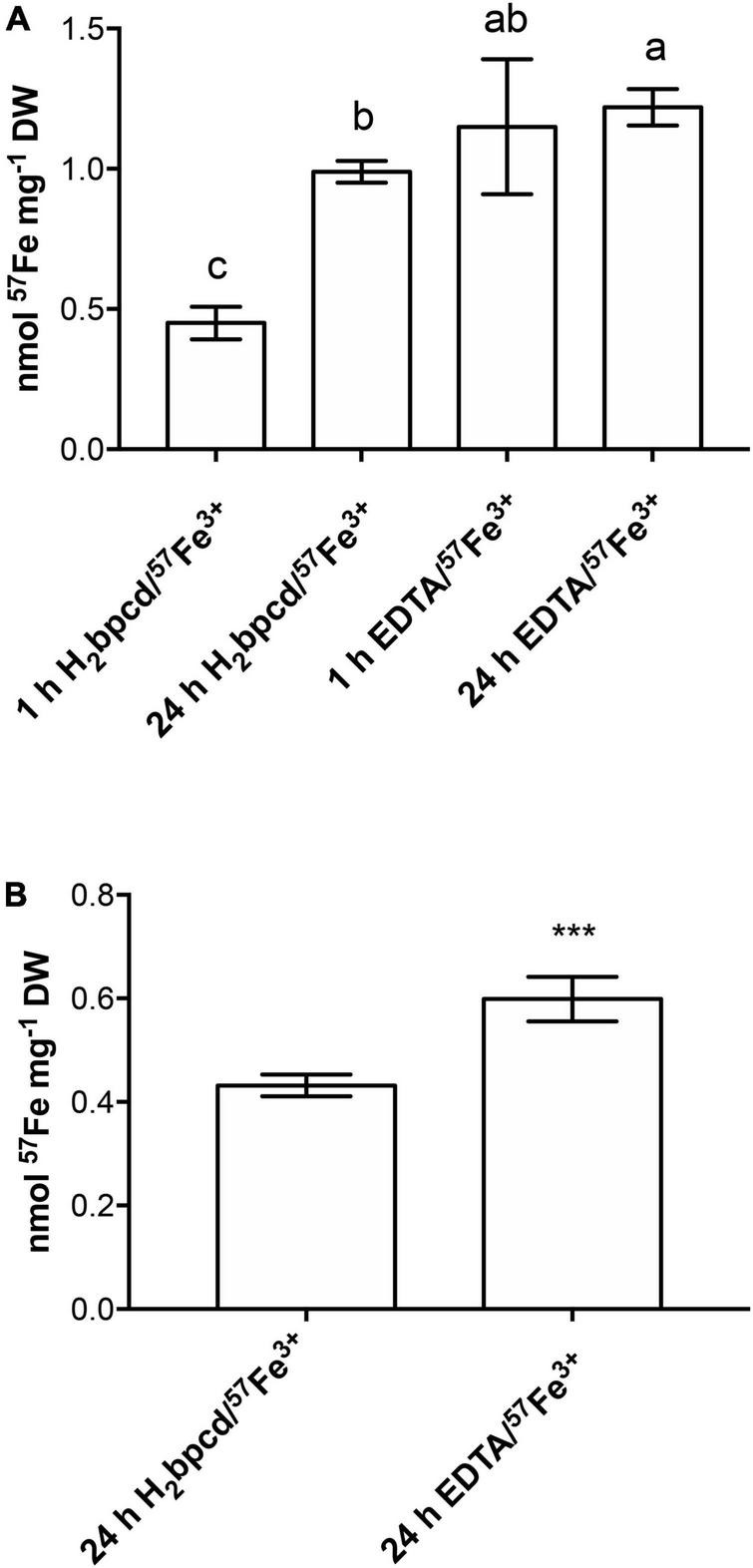
^57^Fe uptake in **(A)** root of cucumber plants after 1 and 24 h of treatment with H_2_bpcd/^57^Fe^3+^ or EDTA/^57^Fe^3+^ and **(B)** in shoot after 24 h of the same treatments. Data represent mean ± S.D. of three independent replicates with two plants each (*n* = 6) (one-way ANOVA with Turkey’s *post hoc* test, *p* < 0.05, significant differences are indicated by different letters and Student’s *t*-test: ***p < 0.001).

H_2_bpcd/^57^Fe^3+^-treated roots showed an increase in ^57^Fe accumulation from 1 h to 24 h, whereas no differences were observed between 1 and 24 h in the case of EDTA/^57^Fe^3+^-treated roots (Figure 8A). In the case of shoot, ^57^Fe was detectable only after 24 h with higher significant values of accumulation for the plants treated with EDTA/^57^Fe^3+^ (Figure 8B). In contrast, the shoot/root Fe content ratio at 24 h exhibited similar values (0.47 for H_2_bpcd/^57^Fe^3^ *vs.* 0.53 for EDTA/^57^Fe^3+^) between the plants supplied with the two Fe-chelates.

## Discussion

The high mobility in the soil of Fe-chelates hinders their use in agriculture, as they lead to a low persistence for the subsequent plant uptake and environmental risk. In the work detailed herein, we studied the behavior of a new Fe-chelate, H_2_bpcd/Fe^3+^, with respect to soil. Furthermore, we investigated how H_2_bpcd/Fe^3+^ can support the growth of Fe-deficient plants of cucumber and supply Fe in hydroponics in the presence of CaCO_3_.

We found that the complex of Fe with bpcd ([Fe(bpcd)]^+^; logβ = 20.86) features a lower stability than that with EDTA (logβ = 25.10) (Delgado et al., 1997), *o,o*-EDDHA(logβ = 35.09) (Yunta et al., 2003), and CDTA (logβ = 29.05, T = 25°C) (Brausam et al., 2009), and a higher one than that with dicarboxylic analog (ethylenediamine-N,N′-diacetic acid, EDDA logβ = 15.5) (Serratrice et al., 2001). This behavior indicates that all donor groups are involved in the binding of Fe^3+^ ion, yet with lower stability than that estimated by Lacoste et al. (1965) with the similar ligand ethylenebis- N,N′-(2-aminomethyl)- pyridine-N,N′-diacetic acid (EDAMPDA, logβ > 21.5).

The two H_2_bpcd/Fe^3+^ and *o*,*o*-EDDHA/Fe^3+^ systems, which were obtained in the presence of solid CaCO_3_, Fe(OH)_3_, and dissolved CO_2_, exhibited similar behavior, as per the speciation data (Supplementary Figure 3 and Table 1). Unlike EDTA, whose higher affinity for Ca^2+^ causes the precipitation of Fe hydroxide, both chelates can specifically maintain Fe^3+^ ion in solution in basic conditions.

The experiments conducted in columns containing soil with a total limestone level of 10.9% confirmed the high mobility of *o*,*o*-EDDHA/Fe^3+^ along the soil profile (Figure 5). In fact, the highly stable *o*,*o*-EDDHA/Fe^3+^ chelate, which should weakly interact with the soil, is highly concentrated in the soil solution and almost fully eluted (about 93%). Similar percentages for the elution of [^59^Fe(EDDHA)]^–^ from the soil columns were reported by Cesco et al. (2000). Conversely, the less stable H_2_bpcd/Fe^3+^ chelate is retained by the soil and provides only a low quantity of Fe^3+^ (no more than 10% of the total applied Fe) after the elution of the same solution volume (Figure 5 and Table 1).

We tentatively rule out the involvement of the attractive coulombic interaction by the negatively charged clay surface, despite the complexity of the mechanisms at the basis of soil/chelate interaction. The analysis and review of these mechanisms exceed the scope of the present contribution, as the dominant species at pH ≥ 7 is bis-hydroxo complex ([Fe(bpcd) (OH)_2_] ^–^; Figure 4), which is not cationic, yet anionic. Furthermore, no significant change in the eluted quantity of H_2_bpcd/Fe^3+^ was observed during the leaching experiment with added Ca^2+^ to the simulated elution solution (Figure 5C). Nevertheless, we may infer that the donating atoms of H_2_bpcd hold responsibility for the interaction with the metal cations present in the soil. Likewise, Hernández-Apaolaza and Lucena (2001) reported similar results for the complex montmorillonite-Ca-Fe-chelate. In addition, given the presence of two OH groups in the [Fe(bpcd)H_–2_] ^–^ complex, the participation of hydrogen bonds in the chelate/soil interaction cannot be indeed neglected.

The results detailed herein suggest that the H_2_bpcd/Fe^3+^ complex can be less leached out of the root zone and that Fe bioavailability might last longer. Such an attitude can avoid frequent Fe replenishment of the rhizosphere to correct the plant deficiency is less frequently required, thereby preventing extensive groundwater pollution and diminishing the concern associated with the use of Fe-chelates (Abadía et al., 2011).

The feasibility of H_2_bpcd/Fe^3+^ as an environmentally friendly alternative to alternative Fe chelator requires the evaluation of its effects on plants. Germination tests underlined that the H_2_bpcd ligand did not influence the seed germination more negatively than EDTA. Given that, we decided to evaluate the ability of H_2_bpcd/Fe^3+^ to supply Fe to Fe-deficient cumber plants in hydroponics. We select the harshest condition for the Strategy I plant, namely, high pH due to the CaCO_3_ presence. The effect of H_2_bpcd/Fe^3+^ treatment was compared to that of EDDHA/Fe^3+^ supplied at three different suboptimal Fe concentrations (0.2, 2, and 20 μM). As expected, the recovery from Fe deficiency was found to be concentration-dependent and faster at 20 μM Fe concentration (Figure 6). At a consistently lower concentration than that is optimal for Fe concentration in hydroponics solution (Agnolon et al., 2001), H_2_bpcd/Fe^3+^- and EDDHA/Fe^3+^-treated plants did not display statistically significant differences in leaf SPAD index, shoot and root dry biomass, and total root length (Supplementary Table 2). Santos et al. (2016) found that the complex between the ligand *tris*(3-hydroxy-4-pyridinonate) and Fe^3+^ improves the plant growth if compared to 20 μM commercial EDDHA/Fe^3+^ supplied to Fe-deficient *Glycine max* plants grown in hydroponics. However, these experiments were carried out at pH 5.5, a value lower than that of CaCO_3_-containing soils where lime-induced chlorosis frequently occurs. Differences between the two Fe sources were observed only at the lower concentrations (0.2 and 2 μM, Supplementary Table 2). In these conditions, EDDHA/Fe^3+^ exhibited an enhanced capacity in supplying the micronutrient, as demonstrated by all the parameters herein evaluated (Supplementary Tables 2, 4).

Comparable values of root FCR activity to those measured in the positive control (Fe-sufficient plants grown at 50 μM) (Figure 6F) confirm effective recovery from the deficiency at 20 μM of both Fe sources. The highest FCR activity was recorded at 0.2 and 2 μM for plants treated with H_2_bpcd/Fe^3+^ and 0.2 μM for EDDHA/Fe^3+^, respectively. Therefore, it can be inferred that the supply of these quantities of Fe was not sufficient for the plants, thereby maintaining an active FCR (Agnolon et al., 2001). Such values were even higher than those measured in the negative controls, which comprised plants grown for 14 days without Fe. The lower levels of FCR activity in the negative control could be linked to the complete absence of the micronutrient. It was reported that the presence of at least some amount of Fe is of the utmost importance to the functioning of FCR (Robinson et al., 1999). Furthermore, the evidence of higher FCR activity in the H_2_bpcd/Fe^3+^-treated plants than those supplied with EDDHA/Fe^3+^ at 2 μM of Fe (Figure 6F) reinforces the hypothesis that the regreening capacity of this new chelator occurs at a concentration as high as 2 μM. Conversely, the shoot Fe content of the plants supplied with 20 μM EDDHA/Fe^3+^ was found to be higher than that measured for the H_2_bpcd/Fe^3+^-treated plants, yet without significant differences in SPAD index (Figures 6A,B, 7F). In addition, we observed a general decrease in the concentration of Ca, K, Mg, Cu, Mn, and Zn after 7 days of supply with both Fe-chelates (Figure 7). A decrease in the leaf content of K, Ca, Cu, Mn, and Zn in cucumber plants was observed during the recovery from Fe deficiency when supplied with different natural Fe complexes (Tomasi et al., 2014). The explanation for the highest content of these nutrients in tissues may be twofold. First, an alteration in cation/anion uptake due to enhanced activity of FCR and Fe transporter which can also uptake other metals. Second, a concentration due to a reduction of shoot dry biomass under deficiency (Tomasi et al., 2014). The pattern of the concentrations of these cationic elements in H_2_bpcd/Fe^3+^- and EDDHA/Fe^3+^-treated plants further confirms that the recovery from Fe deficiency started earlier for the second chelate.

In the end, we discussed the possible differences occurring in the uptake of Fe by roots during the recovery in the suboptimal range of concentration (2 μM) using the ^57^Fe as a tracer chelated to H_2_bpcd and EDTA. When treated with EDTA/^57^Fe^3+^ rather than with H_2_bpcd/Fe^3+^ (Figure 8), roots exhibited a significantly higher content of ^57^Fe after 1 h and 24 h, and shoots after 24 h. The correlation between chelate characteristics (charge and logβ), FCR activity, and the subsequent Fe uptake has not been fully explained yet (Chaney, 1989; Lucena and Chaney, 2006). Lucena (2006) asserted that the weakest Fe^3+^-chelates consist in the best substrates for the FCR in Fe-deficient cucumber roots. H_2_bpcd/Fe^3+^ should, indeed, represent a better substrate than both EDTA/Fe^3+^ and EDDHA/Fe^3+^. The following points may support the rationale for such a seeming contradiction: (i) the lower availability for the substrate of the enzyme caused by a potential stronger interaction of the H_2_bpcd/Fe^3+^ molecule with the cell wall; (ii) the stability of the H_2_bpcd/Fe^2+^ counterparts, as the Fe uptake involves Fe^2+^, and (iii) the strong and likely crucial impact on the release of the metal ion in solution produced by the kinetic inertness of the metal complexes (quite often neglected). It is widely recognized that the presence of the *trans*-1,2-diaminocyclohexane ring in the ligand backbone can originate a rigidifying effect, thereby increasing the kinetic stability of the related metal complex. This holds true for lanthanide(III)-based complexes (Tircsó et al., 2016), which is also observed for Fe-chelates. Therefore, it can be speculated that the H_2_bpcd/Fe^3+^ kinetic stability is as high as slow as the Fe supply to plant is. Since the deepening of the biochemical reasons behind the differences in the rate of Fe uptake stretches beyond the scope of this work, we will discuss this aspect in a further paper.

## Conclusion

The obtained data demonstrate that, as hypothesized, the H_2_bpcd/Fe^3+^ complex interacts with soil particles and is much less mobile than EDDHA/Fe^3+^. In hydroponics, the complex, which is even less effective in the regreening than EDDHA/Fe^3+^ at very low Fe concentrations, proved to be capable of restoring the physiological Fe concentration at 20 μM. These two characteristics suggest that the H_2_bpcd/Fe^3+^ complex is a good candidate as new environmentally friendly Fe fertilizer. The validation of the information obtained in simplified laboratory scale experiments demands further studies to be carried out in plant-soil microcosms or in fields.

## Data availability statement

The original contributions presented in this study are included in the article/Supplementary material, further inquiries can be directed to the corresponding author.

## Author contributions

ZV and FP conceived the research. AZ directed the experiments. DS, AM, SR, and MS performed the experiments. FP, DS, AZ, AM, and ZV analyzed and interpreted the data. FP, ZV, and AZ wrote the manuscript. FP, AM, ZV, and AZ revised the manuscript. ZV obtained the funding to carry out the investigation. All authors contributed to the article and approved the submitted version.

## Footnotes

1 For the metal (M) – ligand (L) overall complex formation reaction, considering also protonation equilibria: *iM + jL + kH ⇋ M_*i*_L_*j*_H_*k*_ $\beta_{M\,i\,L\,j\,H\,k}=\frac{\left[M_{\text{i}}\,L_{j}\,H_{k}\right]}{{\left[M\right]}^{i}\,{\left[L\right]}^{j}\,{\left[H\right]}^{k}}$*

2
https://www.eawag.ch/en/department/surf/projects/chemeql/
