# Supplementary material for: Regreening properties of the soil slow-mobile H2bpcd/Fe3+ complex: Steps forward to the development of a new environmentally friendly Fe fertilizer
Source: Front Plant Sci. 2022 Aug 4;13:964088. doi: 10.3389/fpls.2022.964088 (PMC9386293; doi:10.3389/fpls.2022.964088)
Supplement: Supplementary file 1 [file Data_Sheet_1.PDF]

Supplementary Material

Figure S1. HPLC chromatogram of the obtained H_2bpcd ligand and H_2bpcd/Fe^{3+} complex. The intensity of the absorption at 263 nm is reported on the Y axis.

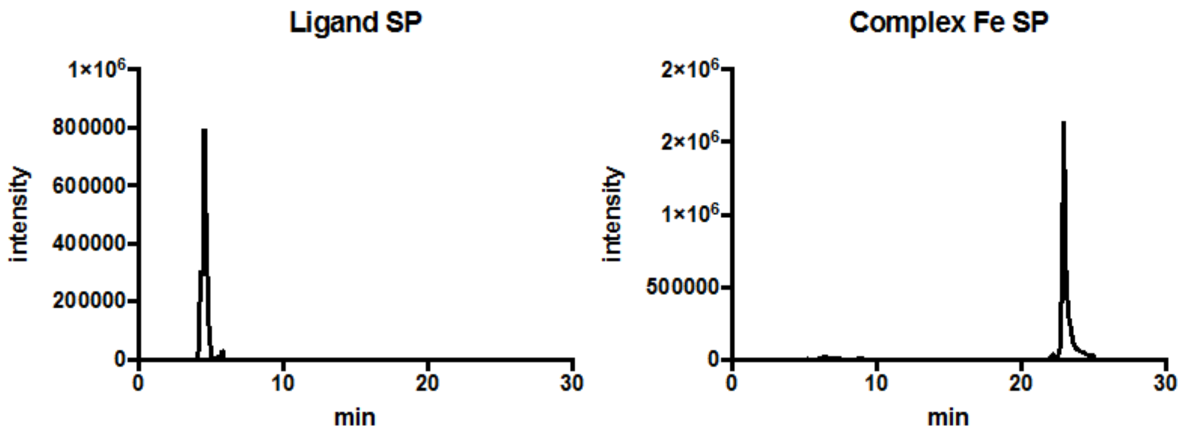

Figure S2. Speciation for the EDTA/Fe^{3+} and EDDHA/Fe^{3+} systems (C_{Fe} = 1.0 mM; 1:1 metal/ligand molar ratio) calculated on the basis of the constants from Delgado et al. (1997). for EDTA and from Yunta et al. (2003) for EDDHA. In the plots charges are omitted for clarity. [Fe(EDTA)] has -1 charge and Fe(EDTA)H_{-1} has -2 charge ($[Fe(EDTA)(OH)]^{2-}$ is the real chemical formula). [Fe(EDDHA)H] is neutral with one protonated phenolic group, $[Fe(EDDHA)]^{-}$ has negative charge and Fe(EDDHA)H_{-1} has -2 charge (its chemical formula is $[Fe(EDDHA)(OH)]^{2-}$).

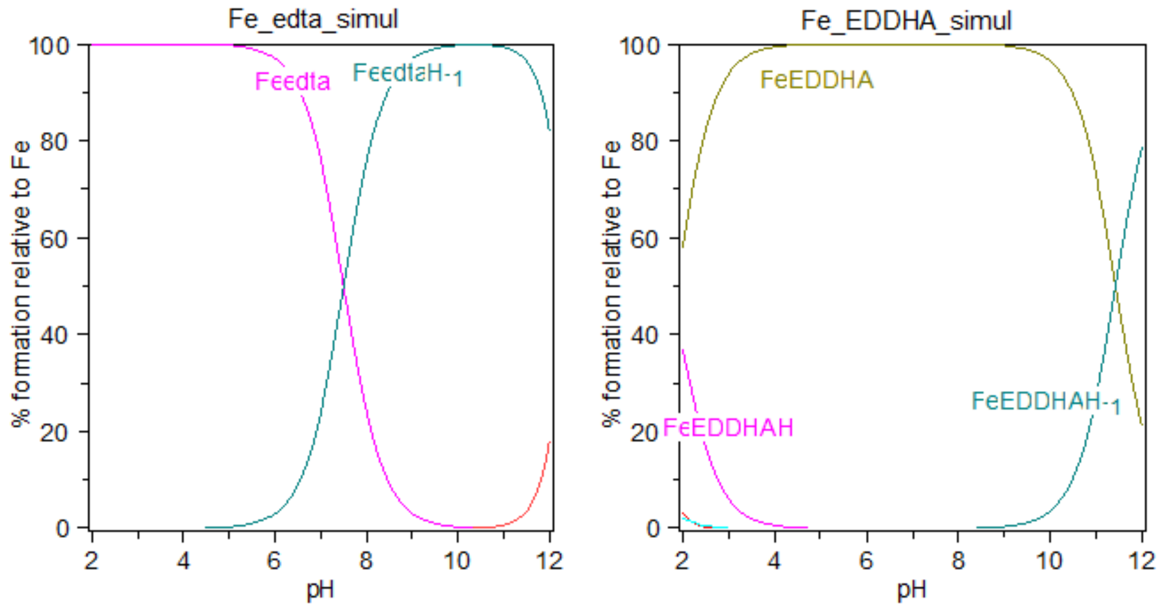

Figure S3. Speciation in solution (log [concentration] *vs.* pH) for the (A) H_2bpcd/Fe^{3+}, (B) EDTA/Fe^{3+} and (C) *o,o*-EDDHA/Fe^{3+} systems (total Fe = total ligand = 2 µM, pH range 2 - 11). Speciation has been calculated considering the presence of dissolved CO_2, solid $CaCO_3$ (calcite) and Fe^{3+} hydrolysis and solubility equilibria. Only the concentrations of the Fe^{3+} species in solution and of the Ca^{2+} complexes with the ligands considered in this work are shown. The vertical red line in (B) marks the pH at which the $Fe(OH)_{3\ (s)}$ precipitation occurs. Charges of the species are omitted for clarity.

A

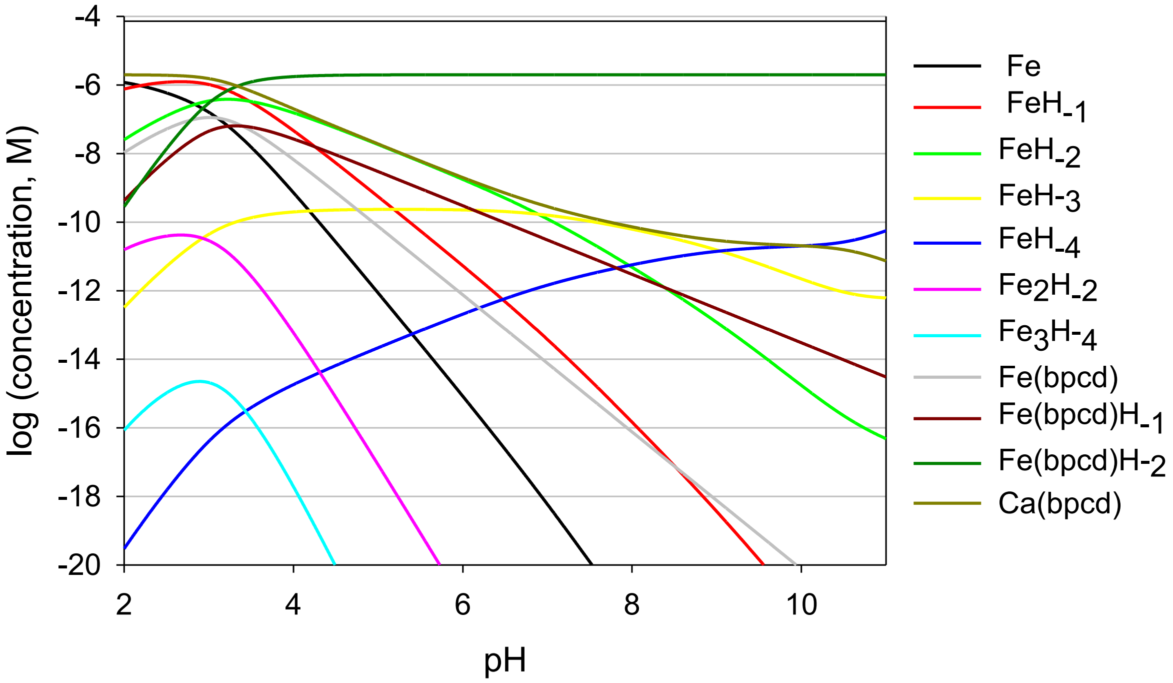

B

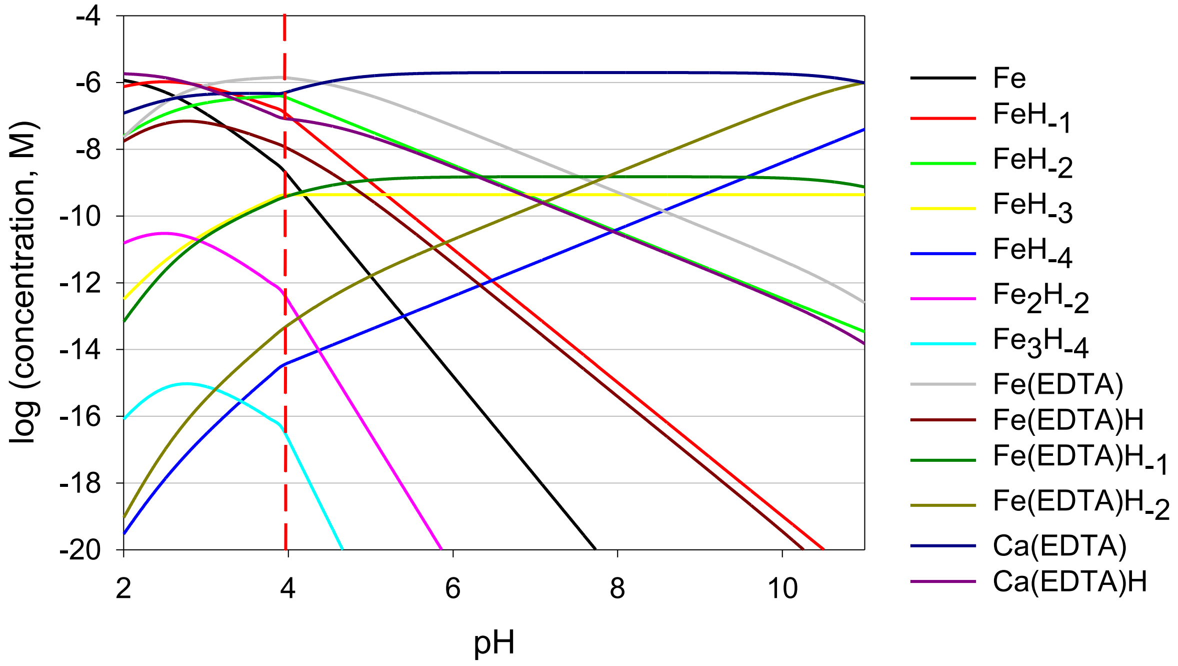

log (concentration, M)
pH
Fe
FeH-1
FeH-2
FeH-3
FeH-4
Fe2H-2
Fe3H-4
Fe(EDTA)
Fe(EDTA)H
Fe(EDTA)H-1
Fe(EDTA)H-2
Ca(EDTA)
Ca(EDTA)H

C

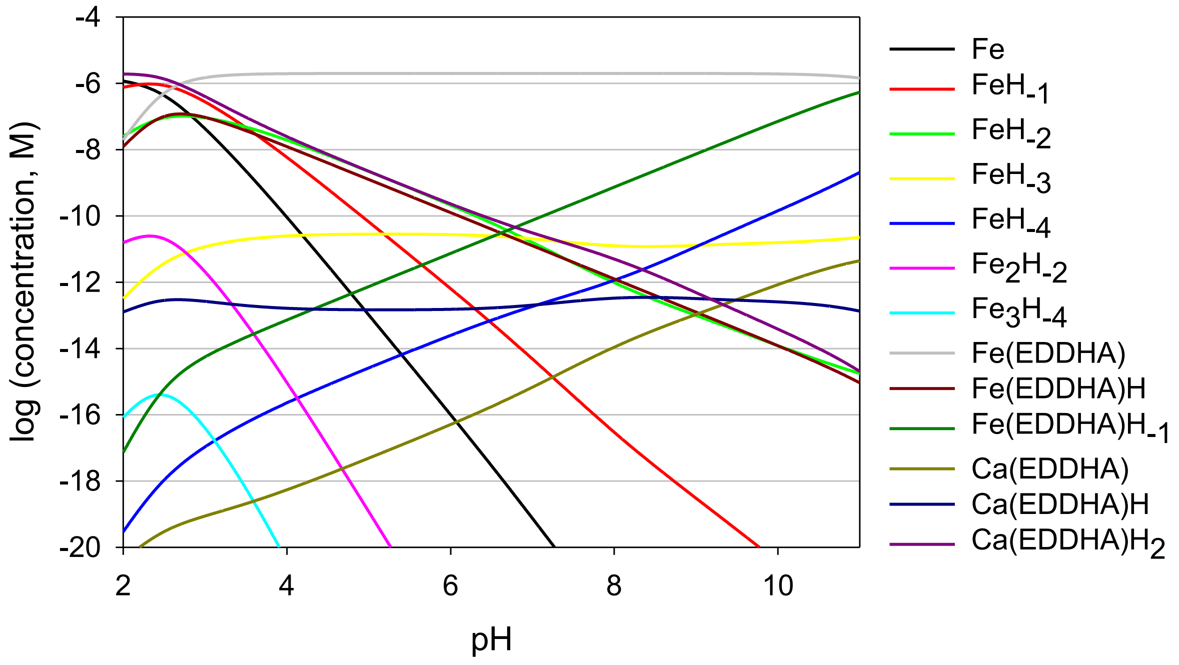

log (concentration, M)
pH
Fe
FeH-1
FeH-2
FeH-3
FeH-4
Fe2H-2
Fe3H-4
Fe(EDDHA)
Fe(EDDHA)H
Fe(EDDHA)H-1
Ca(EDDHA)
Ca(EDDHA)H
Ca(EDDHA)H2

Figure S4. Comparison of leaching profiles through the soil columns of (A) EDDHA/Fe^{3+}eluted with H_2O, (B) H_2bpcd/Fe^{3+} eluted with H_2O and (C) H_2bpcd/Fe^{3+}eluted with 0.5 M $CaCl_2$; (D) Amounts of eluted Fe expressed as percentage of applied Fe. Every single replica is shown.

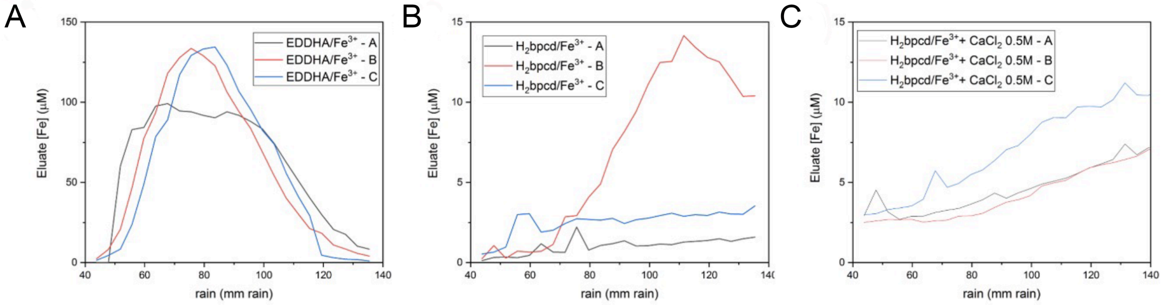

D

	EDDHA/Fe^{3+} +H_2O			H_2bpcd/Fe^{3+} +H_2O			H_2bpcd/Fe^{3+} +$CaCl_2$		
**Replica**	**A**	**B**	**C**	**A**	**B**	**C**	**A**	**B**	**C**
**Total eluted Fe (µmol)**	7.34	7.14	6.95	0.12	0.83	0.30	0.61	0.56	0.92
**Eluted Fe as % of applied Fe**	95.4	92.7	90.3	1.6	10.7	5.4	7.9	7.2	12.0

Figure S5. Cucumber plants during the recovery from Fe deficiency. C+: positive control, C-: negative controls, H_2bpcd/Fe^{3+}: Fe-deficient plants supply with 0.2, 2 and 20 µM of H_2bpcd/Fe^{3+}, EDDHA/Fe^{3+}: Fe-deficient plants supply with 0.2, 2 and 20 µM of EDDHA/Fe^{3+}. T0: plants grown for 7 days with (C+) and without Fe, 1 d: plants after 1 day of supply with H_2bpcd/Fe^{3+} and EDDHA/Fe^{3+}, 2 d: plants after 2 days of supply with H_2bpcd/Fe^{3+} and EDDHA/Fe^{3+}, 6 d: plants after 6 days of supply with H_2bpcd/Fe^{3+} and EDDHA/Fe^{3+}, 7 d: plants after 7 days of supply with H_2bpcd/Fe^{3+} and EDDHA/Fe^{3+}.

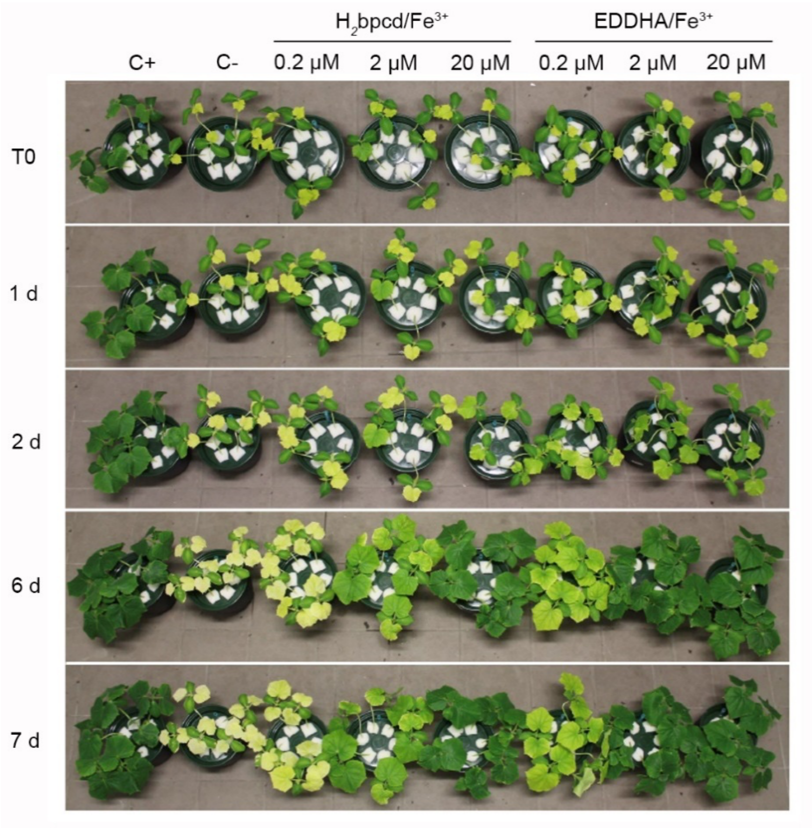

Table S1. SPAD index of cucumber plants at T0 (before resupplying Fe). Data represent means ± S.D. of three independent replicates with four plants each (n=12) (one-way ANOVA with Turkey's post hoc test, $p<0.05$, significant differences are indicated by different letters). A: Fe-deficient plants treated with 0.2 μM H_2bpcd/Fe^{3+}; B: Fe-deficient plants treated with 2 μM H_2bpcd/Fe^{3+}; C: Fe-deficient plants treated with 20 μM H_2bpcd/Fe^{3+}; D: Fe-deficient plants treated with 0.2 μM EDDHA/Fe^{3+}; E: Fe-deficient plants treated with 2 μM EDDHA/Fe^{3+}; F: Fe-deficient plants treated with 20 μM EDDHA/Fe^{3+}.

	**Control**	**-Fe**	**-Fe A**	**-Fe B**	**-Fe C**	**-Fe D**	**-Fe E**	**-Fe F**
**SPAD index**	36.0±1.3 a	6.8±3.0 b	9.2±3.3 b	9.1±3.0 b	8.9±3.5 b	6.4±2.4 b	7.5±6.2 b	7.8±2.7 b

Table S2. Comparison of morpho-physiological parameters between Fe-deficient cucumber plants supplied for 7 days with the two Fe sources applied at the same concentration. Data represent means ± S.D. of three independent replicates with four plants each (n=12) for SPAD index and two plants each (n=6) for other parameters (Student's t-test, * p<0.05; ** p<0.01; *** p<0.001; **** p<0.0001).

	**0.2 µM**		**2 µM**		**20 µM**	
	**H_2bpcd/Fe^{3+}**	**EDDHA/Fe^{3+}**	**H_2bpcd/Fe^{3+}**	**EDDHA/Fe^{3+}**	**H_2bpcd/Fe^{3+}**	**EDDHA/Fe^{3+}**
**SPAD Index 1st leaf**	2.0±0.7 ***	12.6±7.6	16.7±3.3 ****	36.7±2.5	40.0±2.6	39.5±2.6
**SPAD Index 2nd leaf**	2.2±0.6 ***	12.8±7.6	23.4±3.4 ****	35.9±0.7	37.2±0.9	36.4±1.1
**Shoot DW (mg)**	172±16 ****	300±24	296±34 ****	421±17	312±56	366±46
**Root DW (mg)**	24±3 *	32±8	32±5 ****	80±13	80±17	74±12
**Total root length (cm)**	428±44	476±68	590±78 ****	1053±162	1362±62	1442±114
**FCR activity (nmol Fe(II)·g^{-1}FW·h^{-1})**	299.2±77.3 ***	733.9±184.9	716.0±67.7 ****	122.8±51.6	33.1±17.7 *	14.1±3.4

Table S3. Macro- and micronutrient content in root tissues of Fe-deficient cucumber plants supplied with H_2bpcd/Fe^{3+} and EDDHA/Fe^{3+} for 7 days. Data represent means ± S.D. of three independent replicates with two plants each (n=6, one-way ANOVA with Turkey's post hoc test, $p < 0.05$, significant differences are indicated by different letters).

			**H_2bpcd/Fe^{3+}**		
	**C**	**-Fe**	**0.2 µM**	**2 µM**	**20 µM**
			mg g^{-1} DW		
**Ca**	12.88±1.19 b	16.66±3.04 b	23.20±4.19 a	14.90±2.39 b	13.63±0.77 b
**K**	69.63±2.17 a	55.14±3.10 b	57.16±1.51 b	64.79±6.42 a	69.99±3.47 a
**Mg**	2.97±0.67 a	2.49±0.17 ab	2.10±0.19 b	2.19±0.14b	1.97±0.15 b
**P**	2.80±0.28 c	5.29±0.47 a	5.35±0.49 a	5.45±0.38 a	4.27±0.30 b
			µg g^{-1} DW		
**Cu**	5.91±1.45 d	271.4±20.4 a	246.3±31.6 ab	188.9±68.14 b	80.85±10.26 c
**Fe**	171.8±24.31 a	94.34±31.04 b	84.02±4.96 b	103.7±10.4 b	155.4±24.11 a
**Mn**	25.03±2.86 b	37.02±6.44 a	30.62±3.86 ab	25.62±6.67 b	11.40±1.35 b
**Zn**	24.78±5.64 c	76.82±16.29 a	84.43±7.27 a	57.77±12.31 b	32.06±3.28 c
			**EDDHA/Fe^{3+}**		
	**C**	**-Fe**	**0.2 µM**	**2 µM**	**20 µM**
			mg g^{-1} DW		
**Ca**	12.88±1.19 b	16.66±3.04 a	15.21±2.41ab	13.35±1.24 ab	13.01±1.61 b
**K**	69.63±2.17 ab	55.14±3.10 c	68.29±5.28 ab	71.70±1.97 a	63.87±6.68 b
**Mg**	2.97±0.67 a	2.49±0.17 ab	2.21±0.15 b	2.15±0.18 b	2.07±0.28 b
**P**	2.80±0.28 c	5.29±0.47 a	4.91±0.52 a	4.07±0.12 b	3.74±0.37 b
			µg g^{-1} DW		
**Cu**	5.91±1.45 d	271.4±20.4 a	120.8±32.28 b	35.61±6.80 c	29.25±5.08 cd
**Fe**	171.8±24.31 a	94.34±31.04 b	68.21±11.12 b	60.51±8.90 b	151.0±20.25 a
**Mn**	25.03±2.86 b	37.02±6.44 a	17.16±4.78 c	11.58±1.48 c	13.10±1.92 c
**Zn**	24.78±5.64 c	76.82±16.29 a	44.72±5.10 b	31.46±2.43 bc	36.33±3.50 bc

Table S4. Comparison of macro- and micronutrients content in the shoots between Fe-deficient cucumber plants supplied for 7 days with the two Fe sources applied at the same concentration. Data represent means ± S.D. of three independent replicates with two plants each (n=6, Student's t-test: * p<0.05; ** p<0.01; *** p<0.001; **** p<0.0001).

	**0.2 µM**		**2 µM**		**20 µM**	
	**H_2bpcd/Fe^{3+}**	**EDDHA/Fe^{3+}**	**H_2bpcd/Fe^{3+}**	**EDDHA/Fe^{3+}**	**H_2bpcd/Fe^{3+}**	**EDDHA/Fe^{3+}**
			mg g^{-1} DW			
**Ca**	66.91±3.60 **	61.01±2.46	56.23±3.04 **	46.4±5.51	49.12±5.15 *	43.63±2.55
**K**	54.67±12.64	42.23±10.29	43.84±4.47 **	27.68±7.15	30.15±2.46	31.09±4.82
**Mg**	12.23±0.54 ***	9.61±0.69	10.49±0.56****	6.47±0.93	7.14±0.89 **	5.17±0.58
**P**	7.18±1.69 **	4.21±1.03	4.49±0.74 **	3.30±0.64	4.00±0.69	3.17±0.65
			µg g^{-1} DW			
**Cu**	21.58±1.19 ****	13.48±1.12	17.06±2.69 ***	8.83±2.70	13.01±1.67 *	9.06±2.80
**Fe**	30.32±3.27	38.64±12.42	46.93±4.93 **	64.88±8.10	67.47±12.17 ***	152.6±26.87
**Mn**	82.65±11.63 ***	45.34±6.65	47.07±6.17 *	33.53±8.84	44.32±6.81	39.21±5.97
**Zn**	60.25±10.27 **	36.00±8.19	44.03±7.63	37.23±7.00	24.26±6.98 **	40.73±7.10

Table S5. Comparison of macro- and micronutrients content in the roots between Fe-deficient cucumber plants supplied for 7 days with the two Fe sources applied at the same concentration. Data represent means ± S.D. of three independent replicates with two plants each (n=6, Student's t-test: * $p<0.05$; ** $p<0.01$; *** $p<0.001$; **** $p<0.0001$).

	**0.2 µM**		**2 µM**		**20 µM**	
	H_2**bpcd/Fe**$^{3+}$	EDDHA/Fe^{3+}	H_2**bpcd/Fe**$^{3+}$	EDDHA/Fe^{3+}	H_2**bpcd/Fe**$^{3+}$	EDDHA/Fe^{3+}
			mg g^{-1} DW			
Ca	23.20±4.19 **	15.21±2.41	14.90±2.39	13.35±1.24	13.63±0.77	13.01±1.61
K	57.16±1.51 **	68.29±5.28	64.79±6.42 *	71.70±1.97	69.99±3.47	63.87±6.68
Mg	2.10±0.19	2.21±0.15	2.19±0.14	2.15±0.18	1.97±0.15	2.07±0.28
P	5.35±0.49	4.91±0.52	5.45±0.38 ***	4.07±0.12	4.27±0.30 *	3.74±0.37
			µg g^{-1} DW			
Cu	246.3±31.6 ****	120.8±32.28	188.9±68.14 **	35.61±6.80	80.85±10.26 ****	29.25±5.08
Fe	84.02±4.96 **	68.21±11.12	103.7±10.4 ****	60.51±8.90	155.4±24.11	151.0±20.25
Mn	30.62±3.86 ***	17.16±4.78	25.62±6.67 **	11.58±1.48	11.40±1.35	13.10±1.92
Zn	84.43±7.27 ****	44.72±5.10	57.77±12.31 **	31.46±2.43	32.06±3.28	36.33±3.50
